# Author Correction: Human SARS-CoV-2 challenge uncovers local and systemic response dynamics

**DOI:** 10.1038/s41586-024-07838-7

**Published:** 2024-08-01

**Authors:** Rik G. H. Lindeboom, Kaylee B. Worlock, Lisa M. Dratva, Masahiro Yoshida, David Scobie, Helen R. Wagstaffe, Laura Richardson, Anna Wilbrey-Clark, Josephine L. Barnes, Lorenz Kretschmer, Krzysztof Polanski, Jessica Allen-Hyttinen, Puja Mehta, Dinithi Sumanaweera, Jacqueline M. Boccacino, Waradon Sungnak, Rasa Elmentaite, Ni Huang, Lira Mamanova, Rakesh Kapuge, Liam Bolt, Elena Prigmore, Ben Killingley, Mariya Kalinova, Maria Mayer, Alison Boyers, Alex Mann, Leo Swadling, Maximillian N. J. Woodall, Samuel Ellis, Claire M. Smith, Vitor H. Teixeira, Sam M. Janes, Rachel C. Chambers, Muzlifah Haniffa, Andrew Catchpole, Robert Heyderman, Mahdad Noursadeghi, Benny Chain, Andreas Mayer, Kerstin B. Meyer, Christopher Chiu, Marko Z. Nikolić, Sarah A. Teichmann

**Affiliations:** 1 Wellcome Sanger Institute, Wellcome Genome Campus, Cambridge, UK; 2 The Netherlands Cancer Institute, Amsterdam, The Netherlands; 3 UCL Respiratory, Division of Medicine, University College London, London, UK; 4 Research Department of Infection, Division of Infection and Immunity, University College London, London, UK; 5 Department of Infectious Disease, Imperial College London, London, UK; 6 Department of Microbiology, Faculty of Science, and Integrative Computational BioScience Center, Mahidol University, Bangkok, Thailand; 7 Ensocell Therapeutics, BioData Innovation Centre, Wellcome Genome Campus, Hinxton, UK; 8 Department of Infectious Diseases, University College London Hospital, London, UK; 9 hVIVO, London, UK; 10 Division of Infection and Immunity, Institute of Immunity and Transplantation, University College London, London, UK; 11 UCL Great Ormond Street Institute of Child Health, London, UK; 12 Theory of Condensed Matter, Cavendish Laboratory, Department of Physics, University of Cambridge, Cambridge, UK; 13 Present Address: Wellcome MRC Cambridge Stem Cell Institute, University of Cambridge, Cambridge, UK

**Keywords:** Viral infection, Computational biology and bioinformatics, RNA sequencing

Correction to: *Nature* 10.1038/s41586-024-07575-x Published online 19 June 2024

In the version of the article initially published, the Acknowledgements was missing the following text, which has now been added to the HTML and PDF versions of the article: “The authors gratefully acknowledge support from the UK Vaccine Taskforce of the Department of Business, Energy and Industrial Strategy of Her Majesty’s Government (BEIS). We thank the following organizations for their invaluable contributions to development and implementation of the SARS-CoV-2 human challenge project: the Royal Free London NHS Foundation Trust, Human Infection Challenge Network for Vaccine Development (HIC-Vac), NIHR Clinical Research Network staff at the Royal Bolton Hospital, and ISARIC4C Investigators (https://isaric4c.net/about/authors/) for providing the clinical material for challenge virus production. C.C. is supported by the NIHR Imperial Biomedical Research Centre (BRC) award to Imperial College Healthcare NHS Trust and Imperial College London. ISARIC4C is funded by the National Institute for Health Research (NIHR; award CO-CIN-01), the Medical Research Council (MRC; grant MC_PC_19059), the NIHR Health Protection Research Unit in Emerging and Zoonotic Infections at University of Liverpool in partnership with Public Health England (PHE), in collaboration with Liverpool School of Tropical Medicine and the University of Oxford (NIHR award 200907), Liverpool Experimental Cancer Medicine Centre provided infrastructure support for this research (grant reference C18616/A25153) and NIHR Health Protection Research Unit in Respiratory Infections (NIHR award 200927). The views expressed are those of the authors and not necessarily those of the NHS, the NIHR, DHSC or BEIS.”

