# Supplementary material for: Human SARS-CoV-2 challenge uncovers local and systemic response dynamics
Source: Nature. 2024 Jun 19;631(8019):189–98. doi: 10.1038/s41586-024-07575-x (PMC11222146; doi:10.1038/s41586-024-07575-x)
Supplement: Supplementary file 1 — Additional findings covering myeloid cell dynamics throughout infection, non-productive SARS-CoV-2 infection of immune cells, hyperinfected ciliated cells, atypical γδ T cell infiltration, B cell clonal expansion and use of this data as a reference atlas. [file 41586_2024_7575_MOESM1_ESM.docx]

## **1. Temporal reduction of myeloid subsets immediately after viral exposure**

To investigate the potential role of professional antigen presenting cells in the early immune response to SARS-CoV-2, we next focused on changes in the nasopharyngeal resident and circulating myeloid compartments during sustained infection. In contrast to most tissue-resident immune cells, myeloid subsets largely decreased in frequency at the site of inoculation by day 3 after inoculation (**Extended Data Fig. 5i, Extended Data Fig. 7f**). In particular, monocytes and activated DCs (also often referred to as migratory DCs, mature DCs or DC-LAMPs) were significantly reduced at the site of inoculation at day 3, consistent with the known role of activated DCs in trafficking viral antigens to lymph nodes for presentation. The decrease in nasopharyngeal monocytes seen in sustained infections was also observed in abortive infections, but not transient infections. After day 3, the number of DCs at the site of inoculation increased above baseline, along with the global immune infiltration associated with peak viral load in sustained infections. We also observed a decrease of circulating myeloid cells during infection across all subsets at day 7, consistent with continued migration of these cells into inflamed or lymphoid tissues at that time point. However, already at day 3 post-inoculation there are strong decreases in some circulating myeloid subsets, where inflammatory monocytes (*IL1B, IL6* & *CXCL3* high) and cDC3s (monocyte-like dendritic cells) appear to migrate out of the circulation.

##

## **2. Non-productive SARS-CoV-2 infection of immune cells**

Having identified infected nasopharyngeal cells, we next asked if these represented productive infections. Because SARS-CoV-2 has a polyadenylated ssRNA genome, we were able to detect both viral transcripts and genomes, which allowed us to separate non-productive and productive infections. In non-productive infections only viral genomes would be present, leading to a fairly uniform distribution of detected viral RNAs over the length of the viral genome (slightly 5’ biased due to 5’ tag sequencing). In contrast, a productive infection requires viral transcription which is known to be highly biased towards the 3’ end of the viral genome[^1^](https://paperpile.com/c/QZKEFw/Ew1Tx). We observed that the viral RNA found in infected ciliated, hyper-infected ciliated, and goblet cells mainly originated from the 3’ end where most genes are encoded, while viral RNA was uniformly distributed across the genome in infected CD8+ T cells and macrophages (**Extended Data Fig. 6d**). This suggests that immune cells are not permissive for or are capable of preventing viral transcription and subsequent replication after viral entry, while goblet and ciliated cells are susceptible to proliferative viral infection. While the detection of inactive SARS-CoV-2 in macrophages could be a consequence of the engulfment of virions and infected cells, it is unclear how infection of tissue-resident CD8+ T cells is achieved and how this affects their function. While the presence of SARS-CoV-2+ CD8+ T cells have been observed in previous single cell RNA-seq studies by us and others[^2,3^](https://paperpile.com/c/QZKEFw/oeqR+JQiy), their infection productivity has never been assessed. We sought to investigate if these infected subpopulations could be in part explained by dynamic viral entry gene expression. Quantifying the expression of viral entry genes reveals that ciliated cells express the highest amount of viral entry genes, which could explain their relatively high infection rate (**Extended Data Fig. 6a**). While infected and non-infected ciliated subsets have relatively homogenous *ACE2* expression, infected secretory cells have higher expression of *ACE2* compared to non-infected secretory cells. In contrast, *ACE2* and *TMPRSS2* expression cannot explain the observed SARS-CoV-2+ myeloid and T cells. In line with this, ACE2-independent SARS-CoV-2 infection of T cells has been observed *in vitro*[*^4^*](https://paperpile.com/c/QZKEFw/d6dB).

##

## **3. Hyper-infected ciliated cells produce anti-inflammatory molecules**

To investigate how the varying amounts of virus per cell affects host cell gene expression, and *vice versa*, we correlated the amount of viral RNA with the expression of host genes. This revealed that ciliated cells exhibited a unique response to high viral amounts, upregulating AP1 and NFKB signaling, and multiple genes with known anti-inflammatory functions such as *ERG1*[^5^](https://paperpile.com/c/QZKEFw/vhXzA), *NFKBIA*[^6^](https://paperpile.com/c/QZKEFw/Gs1df), *GDF15*[^7^](https://paperpile.com/c/QZKEFw/gUZH8), *HES1*[^8^](https://paperpile.com/c/QZKEFw/3cnLs), *PER1*[^9^](https://paperpile.com/c/QZKEFw/MADjy), *TNFAIP3*[^10^](https://paperpile.com/c/QZKEFw/M8PRh), and *NR4A1*[^11^](https://paperpile.com/c/QZKEFw/DccRz) (**Extended Data Fig. 6c**). This suggests that SARS-CoV-2 is associated with a unique response state in hyper-infected ciliated cells that is in part anti-inflammatory. This together with the attenuation of antigen presentation machinery and the interferon, APR, and innate responses in hyper-infected ciliated cells possibly enhances viral spread and survival (**Extended Data Fig. 5g and 6c**).

##

## **4. Atypical γδ T cells infiltrate site of infection and dominate the γδ T cell response**

In the nasopharynx of sustained infections, we also detected a subset of CD4+ and CD8+ T cells lacking both activation and tissue-residency markers (such as *ITGAE,* **Extended Data Fig. 3a**) which appeared in the nasopharynx during sustained infections and which were annotated as “infiltrating memory T cells” (**Fig. 3e**). We noticed that infiltrating CD8+ T cells expressed relatively few detectable αβ TCRs and had heterogenous CD8 expression, similarly to the γδ T cells that we had already detected (**Extended Data Fig. 2a**). To investigate if this infiltrating subset harbors a distinct γδ T cell population, we performed targeted single cell sequencing of the γδ TCR genes in the nasopharynx and blood. This revealed that infiltrating CD8+ T cells indeed predominantly express γδ TCRs (**Extended Data Fig. 7a**). As expected, the γδ TCR repertoire found in circulating blood cells consists mostly of TCR chains containing variable segments TRDV2 and TRGV9 (**Extended Data Fig. 7b**). Strikingly, the nasopharynx is significantly depleted for TRDV2/TRGV9+ T cells, with other variable segments dominating the γδ TCR repertoire. More than 97% of the γδ TCR expressing infiltrating CD8+ T cells express these rare non-TRDV2/TRGV9 TCRs (which we termed atypical γδ T cells; (**Extended Data Fig. 7a**), which means that the atypical γδ T cell response is four times more abundant than the typical γδ T cell response. While the exact function of atypical γδ T cells is still poorly understood, their timing alongside other adaptive immune responses and its restriction to sustained infections, suggests that they might play an underappreciated role in the immune response against SARS-CoV-2 infection.

## **5. Antibody secreting B cells clonally expand ten days after exposure**

Given that the strong T cell response that appears highly time restricted to day 10 post-inoculation, we hypothesized that there should be a B cell response at a similar time point. To test this, we investigated the temporal and cell state dynamics of the B cell response to SARS-CoV-2 inoculation. We detected distinct subtypes of naive, memory and antibody-secreting B cells (plasmablasts and plasma cells), and used the BCR data to distinguish immunoglobulin class and isotype switching (**Extended Data Fig. 4b**). In line with the observed T cell response, we observe a strong and highly time restricted B cell response from day 10-14 after SARS-CoV-2 exposure in sustained infections (**Extended Data Fig. 7d**). In blood, this response includes a clear switch from naive and IgG/IgA memory B cells to mostly IgG1 and some IgA1 secreting plasmablasts and plasma cells. IgA1 and IgG1 are expected to be the dominant antibody immunoglobulin classes in blood[^12^](https://paperpile.com/c/QZKEFw/wqg9e), and the timing of production of antibodies is in line with B cell responses observed in vaccination studies[^13^](https://paperpile.com/c/QZKEFw/oshxm), suggesting that these antibody secreting B cells at day 10 after inoculation are SARS-CoV-2 specific. While numbers of detected B cells in the nasopharynx are limited, we also observe significant infiltration of both IgA+ and IgG+ B cells into the nasopharynx from day 10 post-inoculation (**Extended Data Fig. 7d,f**), indicating that the B cell response leads to antibody production at the site of infection. No such changes were observed in the nasopharynx or blood of abortive infections, likewise with the blood of transient infections (**Extended Data Fig. 7e-g**). However, as noted previously, a global and immediate infiltration of the B cell compartment was seen in the nasopharynx of transient infections, with a smaller secondary wave at day 10 post-inoculation, suggesting that these patients still mounted a limited B cells response, although restricted to the local site of inoculation. Together, these findings suggest that it takes ten days from SARS-CoV-2 exposure for the adaptive immune response to mature and expand to detectable abundances. Importantly, we show a concerted adaptive immune response of B and T cells at both local and systemic level, which is facilitated by antibody secreting and activated lymphocytes.

##

## **6. Human COVID-19 challenge data as a reference atlas for cell dynamics**

To maximize the impact of our time-resolved COVID-19 dataset, we build predictive models to infer time since SARS-CoV-2 exposure. We used Gaussian process regression and latent variable models to fit the changes in cell state composition during sustained infection. We next applied these predictions to publicly available PBMC single cell RNA-seq datasets from 361 COVID-19 samples, to infer at which stage of the immune response each patient was and to predict when this patient was exposed to SARS-CoV-2. Reassuringly, our Gaussian processes based time inference model predicts that the time since exposure and the time since onset of symptoms are highly correlated (**Extended Data Fig. 8f**), and that exposure is predicted to precede onset of symptoms, as expected. Interestingly, the predicted difference between exposure and symptoms decreases with increased severity (**Extended Data Fig. 6o**), where patients with more severe COVID-19 are predicted to be in the adaptive immune reaction phase for longer. While this suggests that patients with severe COVID-19 take longer to clear the virus, it could also indicate that the cellular composition and immune response timeline in severe cases is perturbed compared to the relatively mild cases observed in the challenge study. In addition to a temporal model that could improve the assessment of the disease stage of COVID-19 patients, we also provide annotation models for a total of 202 cell states including new temporal and rare cell states. These models are now included in the default models at CellTypist.org, and enable highly detailed cell type annotation without the need for bioinformatics expertise. In addition, our single cell expression data is freely available at our COVID19CellAtlas.org web portal for online exploration and analysis.

##

# **References**

1. [Kim, D. *et al.* The Architecture of SARS-CoV-2 Transcriptome. *Cell* **181**, 914–921.e10 (2020).](http://paperpile.com/b/QZKEFw/Ew1Tx)

2. [Loske, J. *et al.* Pre-activated antiviral innate immunity in the upper airways controls early SARS-CoV-2 infection in children. *Nat. Biotechnol.* **40**, 319–324 (2022).](http://paperpile.com/b/QZKEFw/oeqR)

3. [Yoshida, M. *et al.* Local and systemic responses to SARS-CoV-2 infection in children and adults. *Nature* **602**, 321–327 (2022).](http://paperpile.com/b/QZKEFw/JQiy)

4. [Shen, X.-R. *et al.* ACE2-independent infection of T lymphocytes by SARS-CoV-2. *Signal Transduct Target Ther* **7**, 83 (2022).](http://paperpile.com/b/QZKEFw/d6dB)

5. [Trizzino, M. *et al.* EGR1 is a gatekeeper of inflammatory enhancers in human macrophages. *Science Advances* vol. 7 Preprint at https://doi.org/](http://paperpile.com/b/QZKEFw/vhXzA)[10.1126/sciadv.aaz8836](http://dx.doi.org/10.1126/sciadv.aaz8836) [(2021).](http://paperpile.com/b/QZKEFw/vhXzA)

6. [Li, Q. & Verma, I. M. NF-κB regulation in the immune system. *Nat. Rev. Immunol.* **2**, 725–734 (2002).](http://paperpile.com/b/QZKEFw/Gs1df)

7. [Luan, H. H. *et al.* GDF15 Is an Inflammation-Induced Central Mediator of Tissue Tolerance. *Cell* **178**, 1231–1244.e11 (2019).](http://paperpile.com/b/QZKEFw/gUZH8)

8. [Shang, Y. *et al.* The transcriptional repressor Hes1 attenuates inflammation by regulating transcription elongation. *Nat. Immunol.* **17**, 930–937 (2016).](http://paperpile.com/b/QZKEFw/3cnLs)

9. [Wang, T. *et al.* PER1 prevents excessive innate immune response during endotoxin-induced liver injury through regulation of macrophage recruitment in mice. *Cell Death Dis.* **7**, e2176–e2176 (2016).](http://paperpile.com/b/QZKEFw/MADjy)

10. [Liu, L., Jiang, Y. & Steinle, J. J. TNFAIP3 is anti-inflammatory in the retinal vasculature. *Mol. Vis.* **28**, 124–129 (2022).](http://paperpile.com/b/QZKEFw/M8PRh)

11. [Scholtysek, C., Uderhardt, S., Schett, G. & Krönke, G. NR4A1 modulates the inflammatory response during murine experimental arthritis. *Ann. Rheum. Dis.* **69**, 37–37 (2010).](http://paperpile.com/b/QZKEFw/DccRz)

12. [Phad, G. E. *et al.* Clonal structure, stability and dynamics of human memory B cells and circulating plasmablasts. *Nat. Immunol.* **23**, 1–10 (2022).](http://paperpile.com/b/QZKEFw/wqg9e)

13. [Fink, K. Origin and Function of Circulating Plasmablasts during Acute Viral Infections. *Front. Immunol.* **3**, 78 (2012).](http://paperpile.com/b/QZKEFw/oshxm)
