# Supplementary material for: Human SARS-CoV-2 challenge uncovers local and systemic response dynamics
Source: Nature. 2024 Jun 19;631(8019):189–98. doi: 10.1038/s41586-024-07575-x (PMC11222146; doi:10.1038/s41586-024-07575-x)
Supplement: Supplementary file 2 — Reporting Summary [file 41586_2024_7575_MOESM2_ESM.pdf]

nature portfolio

Corresponding author(s): Sarah A Teichmann
Marko Z Nikolić
Rik GH Lindeboom

Last updated by author(s): Apr 17, 2024

Reporting Summary

Nature Portfolio wishes to improve the reproducibility of the work that we publish. This form provides structure for consistency and transparency in reporting. For further information on Nature Portfolio policies, see our Editorial Policies and the Editorial Policy Checklist.

Statistics

For all statistical analyses, confirm that the following items are present in the figure legend, table legend, main text, or Methods section.

n/a	Confirmed	
☐	☒	The exact sample size (*n*) for each experimental group/condition, given as a discrete number and unit of measurement
☐	☒	A statement on whether measurements were taken from distinct samples or whether the same sample was measured repeatedly
☐	☒	The statistical test(s) used AND whether they are one- or two-sided<br>*Only common tests should be described solely by name; describe more complex techniques in the Methods section.*
☐	☒	A description of all covariates tested
☐	☒	A description of any assumptions or corrections, such as tests of normality and adjustment for multiple comparisons
☐	☒	A full description of the statistical parameters including central tendency (e.g. means) or other basic estimates (e.g. regression coefficient) AND variation (e.g. standard deviation) or associated estimates of uncertainty (e.g. confidence intervals)
☐	☒	For null hypothesis testing, the test statistic (e.g. *F*, *t*, *r*) with confidence intervals, effect sizes, degrees of freedom and *P* value noted<br>*Give P values as exact values whenever suitable.*
☒	☐	For Bayesian analysis, information on the choice of priors and Markov chain Monte Carlo settings
☒	☐	For hierarchical and complex designs, identification of the appropriate level for tests and full reporting of outcomes
☐	☒	Estimates of effect sizes (e.g. Cohen's *d*, Pearson's *r*), indicating how they were calculated

Our web collection on statistics for biologists contains articles on many of the points above.

Software and code

Policy information about availability of computer code

Data collection	Single-cell RNA-seq and CITE-seq data from PBMCs was jointly aligned against the GRCh38 reference that 10X Genomics provided with CellRanger 3.0.0, and alignment was performed using CellRanger 4.0.0. CITE-seq antibody-derived tag (ADT) barcodes were aligned against a barcode reference provided by the supplier, which we annotated to add informative protein names and made available in our GitHub repository. Single-cell RNA-seq data from nasopharyngeal swab samples were aligned against the same reference using STARSolo 2.7.3a, and post-processed with an implementation of emptydrops extracted from CellRanger 3.0.2. To detect viral RNA in infected cells, we added 21 viral genomes including pre-Alpha SARS-CoV-2 (NC_045512.2) to the above mentioned reference genomes for RNA-seq alignment, as described in Yoshida et al, Nature, 2022. Single cell alpha/betaTCR and BCR data was aligned using CellRanger 4.0.0 with the accompanying GRCh38 VDJ reference that 10X Genomics provided. Single cell gamma/delta TCR data was aligned against the GRCh38 reference that 10X Genomics provided with CellRanger 5.0.0, using CellRanger 6.1.2.
Data analysis	Both single cell RNA-seq and ADT-seq data were corrected using SoupX 1.5.2 (Young and Behjati, 2020) to remove free-floating and background RNAs and ADTs. To correct ADT counts, SoupX 1.5.2 parameters soupQuantile and tfidfMin parameters were set to 0.25 and 0.2, respectively, and lowered by decrements of 0.05 until the contamination fraction was calculated using the autoEstCont function. SoupX on RNA data was performed using default settings. To confidently annotate SARS-CoV-2 infected cells, we used SoupX corrected viral RNA counts to remove false positives due to freely floating SARS-CoV-2 virions. To profile the distribution of viral reads, we removed PCR duplicates from the aligned BAM files that STARSolo produced with MarkDuplicates in picard (https://broadinstitute.github.io/picard/), and tallied the location within the SARS-CoV-2 genome using the start of each sequencing read. Aligned single cell RNA-seq data was imported from the filtered_feature_bc_matrix folder into Seurat V4.1.0 for processing, keeping only cells with at least 200 RNA features detected. Nasopharyngeal and PBMC cells with more than 50% and 10% of the counts coming from mitochondrial genes were excluded, respectively. SoupX corrected gene expression and ADT counts were normalized by dividing it by the total counts per cell and multiplying by 10 000, followed by adding one and a natural-log transformation (log1p).

Each PBMC sample was pooled twice into two unique pools containing up to four PBMC samples per pool, followed by CITE-seq and single cell VDJ sequencing as described above. Souporcell V2.0 (Heaton et al. 2020) was used to demultiplex each pools based on the genotype differences between the mixed samples. Souporcell analyses were performed with the skip_remap parameter enabled and using the common SNP database that was provided by the software. We used two complementary approaches to confidently assign participant identity to each souporcell cluster. First we compared the cluster genotypes with SNP array derived genotyping data, generated for all participants and performed using the Affymetrix UK Biobank AxiomTM Array kit by Cambridge Genomic Services (CGS). Second, the combinations of samples within each pool was unique, enabling assignment of participant identity based on the presence of unique participant-specific combinations of identical genotypes in two separate pools. This multiplexing and replication strategy furthermore enabled us to distinguish library specific batch effects from participant specific effects in downstream analyses.

Aligned single cell BCR and alpha/beta TCR sequencing data was imported in scirpy to obtain a cell by TCR or BCR formatted table, which was then added to Seurat objects containing gene expression data. Aligned single cell gamma/delta TCR data was reannotated using Dandelion V0.2.4. TCR sequences were compared to human SARS-CoV-2 specific entries from https://www.iedb.org/ fetched on 24.08.2023.

All custom code developed in this study is publicly available at: https://github.com/Teichlab/COVID-19_Challenge_Study, with the 'Release for Nature publication' version marking the last commit (90e64cb) before submission.

Other bioinformatics analyses used the following packages with version:

Python: python (3.9.16), ipykernel (6.14.0), numpy (1.23.5), pandas (1.5.3), scanpy (1.9.3), celltypist (1.3.0), tcrdist3 (0.2.2), igraph (0.10.4), leidenalg (0.9.1), matplotlib (3.7.1), seaborn (0.11.2), logomaker (0.8), celltcr (0.1), statannotations (0.5.0), scipy (1.10.1),

R: R (4.0.4), Seurat (4.0.1), tidyverse (1.3.1), ggplot2 (3.3.6), harmony (1.0), ComplexHeatmap (2.6.2), sceasy (0.0.6), reticulate (1.18), SoupX (1.5), rvcheck (0.2.1), cardelino (1.4.0), randomcoloR (1.1.0.1), ggh4x (0.2.8), circlize (0.4.15), readr (1.4.0), lme4 (1.1-29), Matrix (1.3-2), numDeriv (2016.8-1.1), Rsamtools (2.6.0), GenomicAlignments (1.26.0), msigdbr (7.5.1), fgsea (1.28.0), glmmSeq (0.1.1), future (1.21.0), igraph (1.2.6), leiden (0.3.7), ggseqlogo (0.2), patchwork (1.1.1)

For manuscripts utilizing custom algorithms or software that are central to the research but not yet described in published literature, software must be made available to editors and reviewers. We strongly encourage code deposition in a community repository (e.g. GitHub). See the Nature Portfolio guidelines for submitting code & software for further information.

Data

Policy information about availability of data

All manuscripts must include a data availability statement. This statement should provide the following information, where applicable:

- Accession codes, unique identifiers, or web links for publicly available datasets
- A description of any restrictions on data availability
- For clinical datasets or third party data, please ensure that the statement adheres to our policy

The data presented in this study can be explored and analyzed interactively through our COVID-19 Cell Atlas web portal (https://covid19cellatlas.org). The cell state annotation model is available in the CellTypist model repository (https://www.celltypist.org/models) under the name ‘COVID19_HumanChallenge_Blood’. A reference for our Multi Task Gaussian Process Regression model to infer time since viral exposure on PBMC data is available at our GitHub repository (https://github.com/Teichlab/COVID-19_Challenge_Study). The raw sequencing data is available under controlled access at the European Genome-Phenome Archive under accession number EGAD00001012227. Processed bulk RNAseq data is available at ArrayExpress (accession number: E-MTAB-12993). Single-cell count matrices with metadata are available at https://www.covid19cellatlas.org/ as h5ad files.

Human research participants

Policy information about studies involving human research participants and Sex and Gender in Research.

Reporting on sex and gender

No sex- or gender-based analyses were performed. This study is based on 16 participants, which is not an appropriate sample size to confidently look for sex- or gender-related effects.

Population characteristics

Sero-negative (no evidence of COVID-19 infection or previous vaccination) healthy male and female volunteers 18-30 years of age (inclusive) with no known risk factors for severe COVID-19.

Recruitment

Screening of potential participants took place in two stages with an initial screening visit, followed by a study specific remote consultation to go through the full study participant information following adequate time for the informed consent form (ICF) and participation in the study to be considered. Screening visits took place between Day -90 to Day -2. Potential participants were screened under a separate study-specific screening protocol using a screening ICF and advertising material that was approved by the Research Ethics Committee (REC) and Health Research Authority (HRA). Screening activities under the separate screening protocol continued up until subjects sign the study specific consent. Recruitment was done through a number of channels:
• Approved advertising, including social media
• hVIVO volunteer database (Volunteers already registered with any other hVIVO database may be contacted to determine their interest in participating in SARS-CoV-2 research.)
• Referral
• Organic search (e.g. via Google or other search engines)

The participant sample was biased by the age criteria (18-30 years) and requirement to be healthy with no co-morbidities or known risk factors for severe COVID-19 based on clinical history, blood tests and radiology. There was potential self-selection

	bias as participation was voluntary and instigated by the volunteers. Due to these factors, direct extrapolation of the results to young children, older adults, those with pre-existing conditions and minority groups may not be possible.
Ethics oversight	This study was conducted in accordance with the protocol, the Consensus ethical principles derived from international guidelines including the Declaration of Helsinki and Council for International Organizations of Medical Sciences (CIOMS) International Ethical Guidelines, applicable ICH Good Clinical Practice guidelines, applicable laws and regulations. The screening protocol and main study were approved by the UK Health Research Authority – Ad Hoc Specialist Ethics Committee (reference: 20/UK/2001 and 20/UK/0002).

Note that full information on the approval of the study protocol must also be provided in the manuscript.

Field-specific reporting

Please select the one below that is the best fit for your research. If you are not sure, read the appropriate sections before making your selection.

☒ Life sciences ☐ Behavioural & social sciences ☐ Ecological, evolutionary & environmental sciences

For a reference copy of the document with all sections, see nature.com/documents/nr-reporting-summary-flat.pdf

Life sciences study design

All studies must disclose on these points even when the disclosure is negative.

Sample size	No sample size calculation was performed. As these are scarce samples, we collected and analyzed as many samples from the two quarantine groups of participants we had access to, which is how the presented sample size was established.
Data exclusions	No samples were excluded from analysis.
Replication	All available samples from two distinct quarantine groups were analyzed. We analyzed the dataset comparing three infection groups (6, 3 and 7 participants per group), looking at changes in PBMCs and nasopharyngeal swabs over time (13-9 samples per participant, which always included all possible samples we were able to obtain). Each PBMC sample was measured twice, each nasopharyngeal sample was measured once. All attempts at replication were successful.
Randomization	None. Participants were not allocated in groups, but all received the same treatment.
Blinding	Blinding was not relevant as all participants received the same treatment and were not allocated to groups.

Reporting for specific materials, systems and methods

We require information from authors about some types of materials, experimental systems and methods used in many studies. Here, indicate whether each material, system or method listed is relevant to your study. If you are not sure if a list item applies to your research, read the appropriate section before selecting a response.

Materials & experimental systems

n/a	Involved in the study	
☐	☒	Antibodies
☒	☐	Eukaryotic cell lines
☒	☐	Palaeontology and archaeology
☒	☐	Animals and other organisms
☐	☒	Clinical data
☒	☐	Dual use research of concern

Methods

n/a	Involved in the study	
☒	☐	ChIP-seq
☒	☐	Flow cytometry
☒	☐	MRI-based neuroimaging

Antibodies

Antibodies used	For CITE-seq: 137 TotalSeq-C Human Cocktail, V1.0 antibodies (BioLegend, cat. # 99814399905). The reagents that were provided were a pre-diluted commercial panel.<br><br>For Dextramer® SARS-CoV-2 antigen specific enrichment via MACSQuant Tyto cell sorting cells were stained with: anti-human CD14 conjugated to FITC (clone: HCD14 , Biolegend cat. # 325603); anti-human CD3 conjugated to APC (clone: UCHT1, Biolegend cat #300458); PE-dCODE Dextramer® (10x) - Gold, SARS-CoV-2 Multi Allele Panel -XL from Immudex. The latter consists of 44 SARS-CoV-2 antigen specific dCODE™ Dextramer® regents, including a 29 MHC I dCODE Dextramer® reagents (Cat #WA05973dXG PE 50 fBC0587, WA05972dXG PE 50 fBC0588, WB05939dXG PE 50 fBC0589, WB05824dXG PE 50 fBC0590, WC05754dXG PE 50 fBC0591, WD05981dXG PE 50 fBC0592, WD05754dXG PE 50 fBC0593, WF05952dXG PE 50 fBC0594, WF06031dXG PE 50 fBC0595, WH05842dXG PE 50 fBC0596, WB02666dXG PE 50 fBC0597, WI03233dXG PE 50 fBC0598, WA06027dXG PE 50 fBC0599, WA06028dXG PE 50 fBC0600, WA06081dXG PE 50 fBC0601, WA05846dXG PE 50 fBC0602, WA06029dXG PE 50 fBC0603,

WB05948dXG PE 50 fBC0604, WB06025dXG PE 50 fBC0605, WB05762dXG PE 50 fBC0606, WC06082dXG PE 50 fBC0607, WC05978dXG PE 50 fBC0608, WD06030dXG PE 50 fBC0609, WD06083dXG PE 50 fBC0610, WD05982dXG PE 50 fBC0611, WF05984dXG PE 50 fBC0612, WH06032dXG PE 50 fBC0613, WH05888dXG PE 50 fBC0614, WH05879dXG PE 50 fBC0615) plus an additional 15 MHC II dCODE Dextramer® reagents (Cat # FA10157DXG PE 25 FBC0351, FA10160DXG PE 25 FBC0352, FA10161DXG PE 25 FBC0353, FA10162DXG PE 25 FBC0354, FA10164DXG PE 25 FBC0355,FA10165DXG PE 25 FBC0356, FA10167DXG PE 25 FBC0357, FA10168DXG PE 25 FBC0358, FA10169DXG PE 25 FBC0359, FA10170DXG PE 25 FBC0360, FA10171DXG PE 25 FBC0361, FA10172DXG PE 25 FBC0362, FA10173DXG PE 25 FBC0363, FA10175DXG PE 25 FBC0364, FA10002DXG PE 25 FBC0365).

Validation

All antibodies employed were commercial antibodies.

137 TotalSeq-C Human Cocktail, V1.0 antibodies validation:
Proteogenomics quality tested. This panel has been optimized on human PBMCs. Full validation results can be downloaded at the suppliers website: https://www.biolegend.com/en-us/products/totalseq-c-human-universal-cocktail-v1-0-19736

anti-human CD14 conjugated to FITC validation for flow cytometry (FC):
FC quality tested. Each lot of this antibody is quality control tested by immunofluorescent staining with flow cytometric analysis. For flow cytometric staining, the suggested use of this reagent is 5 µl per million cells in 100 µl staining volume or 5 µl per 100 µl of whole blood.
Application References: McMichael A, et al. 1987. Leucocyte Typing III. Oxford University Press. New York.; Knapp W, et al. Eds. 1989. Leucocyte Typing IV. Oxford University Press. New York.; Schlossman S, et al. Eds. 1995. Leucocyte Typing V. Oxford University Press. New York.

anti-human CD3 conjugated to APC validation for flow cytometry (FC):
FC quality tested. Each lot of this antibody is quality control tested by immunofluorescent staining with flow cytometric analysis. For flow cytometric staining using the µg size, the suggested use of this reagent is ≤0.25 µg per million cells in 100 µl volume. It is recommended that the reagent be titrated for optimal performance for each application. For flow cytometric staining using the test sizes, the suggested use of this reagent is 5 µl per million cells in 100 µl staining volume or 5 µl per 100 µl of whole blood.
FC application References: Thakral D, et al. 2008. J. Immunol. 180:7431.; Yoshino N, et al. 2000. Exp. Anim. (Tokyo) 49:97.

Clinical data

Policy information about clinical studies
All manuscripts should comply with the ICMJE guidelines for publication of clinical research and a completed CONSORT checklist must be included with all submissions.

Clinical trial registration

Clinicaltrials.gov NCT04865237

Study protocol

Study protocol is described in Killingley et al, Nature Medicine, 2022.

Data collection

The study was conducted at the Queen Mary BioEnterprises (QMB) Innovation Centre, London, UK (outpatient screening and follow-up visits) and Royal Free London NHS Trust, London, UK (in-patient quarantine). The first date of participant enrollment was 6th March 2021 and the last was 8th July 2021.
Data collection occurred at:
Study specific screening Day -90 to Day -2
Quarantine Phase Day -2 to Day 14 (+ extended days)
Follow up visits Day 28 (+/-3 days), Day 90 (+/- 7 days), Day 180 (+/- 14 days), Day 270 (+/- 14 days) and Day 360 (+/- 14 days)

Outcomes

Primary Objective / Endpoint:
• To identify a safe and infectious dose of wild type SARS-CoV-2 in healthy volunteers, suitable for future intervention studies, that:
• has an acceptable safety profile as measured by:
o Occurrence of Adverse Events (AEs) within 30 days post-viral challenge (Day 0) up to Day 28 follow up.
o Occurrence of Serious Adverse Events (SAEs) from the viral challenge (Day 0) up to Day 28 follow up.
• induces laboratory confirmed infection in ≥50% of participants

Secondary Objectives / Endpoints:

Objective: To further assess SARS-CoV-2 viral infection rates in upper respiratory samples in healthy volunteers, by inoculum dose
Endpoints: To assess the incidence of laboratory confirmed infection rates using a) mid turbinate samples, b) throat swabs, and c) both mid turbinate and throat swabs, as defined by:
• Variant 2: Occurrence of at least two quantifiable (≥LLOQ) RT-PCR measurements, reported on 2 or more consecutive timepoints, starting from 24 hours post-inoculation and up to discharge from quarantine.
• Variant 3: Occurrence of at least two detectable (≥LLOD) RT-PCR measurements, reported on 2 or more consecutive timepoints, starting from 24 hours post-inoculation and up to discharge from quarantine.
• Variant 4: Occurrence of at least one quantifiable (≥LLOQ) SARS-CoV-2 viral cell culture measurement, starting from 24 hours post-inoculation and up to discharge from quarantine.

Objective: To assess the incidence of symptomatic SARS-CoV-2 infection, in healthy volunteers, by inoculum dose
Endpoints: To assess the incidence of lab-confirmed symptomatic SARS-CoV-2 infection using a) mid turbinate samples, b) throat swabs, and c) both mid turbinate and throat swabs, defined as:
• Variant 1:

o Occurrence of at least two quantifiable (≥LLOQ) RT-PCR measurements, reported on 2 or more consecutive timepoints, starting from 24 hours post-inoculation and up to discharge from quarantine, AND
o Either one or more positive clinical symptoms of any grade from two different categories in the symptom scoring system (Upper Respiratory, Lower Respiratory, Systemic), or one Grade 2 symptom from any category
• Variant 2:
o Occurrence of at least two detectable (≥LLOD) RT-PCR measurements, reported on 2 or more consecutive timepoints, starting from 24 hours post-inoculation and up to discharge from quarantine, AND
o Either one or more positive clinical symptoms of any grade from two different categories in the symptom scoring system (Upper Respiratory, Lower Respiratory, Systemic), or one Grade 2 symptom from any category
• Variant 3:
o Occurrence of at least one quantifiable (≥LLOQ) SARS-CoV-2 viral cell culture measurement, starting from 24 hours post-inoculation and up to discharge from quarantine, AND
o Either one or more positive clinical symptoms of any grade from two different categories in the symptom scoring system (Upper Respiratory, Lower Respiratory, Systemic), or one Grade 2 symptom from any category

Objective: To assess the SARS-CoV-2 viral dynamics in upper respiratory samples (AUC, peak, duration, incubation period) in healthy volunteers, by inoculum dose
Endpoints: To assess the viral dynamics using a) mid turbinate samples, and b) throat swabs, as measured by:
• Area under the viral load-time curve (VL-AUC) of SARS-CoV-2 as determined by qRT-PCR, starting from 24 hours post-inoculation and up to discharge from quarantine.
• Peak viral load of SARS-CoV-2 as defined by the maximum viral load determined by quantifiable (≥LLOQ) qRT PCR measurements, starting from 24 hours post-inoculation and up to discharge from quarantine
• Duration of SARS-CoV-2 quantifiable (≥LLOQ) qRT PCR measurements, starting from 24 hours post-inoculation and up to discharge from quarantine. Duration is defined as the time (hours) from the first quantifiable of the two viral quantifiable positives used to assess infection until first confirmed undetectable assessment after their peak measure (after which no further virus is detected).
• Incubation period of SARS-CoV-2 qRT PCR measurements. Incubation period is defined as the time (hours) from inoculation to the first quantifiable of the two viral quantifiable positives used to assess infection, starting from 24 hours post-inoculation and up to discharge from quarantine.
The above endpoints will also be evaluated using quantitative cell culture.

Objective: To assess the SARS-CoV-2 induced symptoms, in healthy volunteers, by inoculum dose
Endpoints:
• Sum total symptoms diary card score: sum total clinical symptoms (TSS) as measured by graded symptom scoring system, starting one day post-viral challenge (Day 1) up to discharge from quarantine
• Area under the curve over time (TSS-AUC) of total clinical symptoms (TSS) as measured by graded symptom scoring system (categorical and visual analogue scales), starting one day post-viral challenge (Day 1) up to discharge from quarantine.
• Peak symptoms diary card score: peak total clinical symptoms (TSS) as measured by graded symptom scoring system (categorical and visual analogue scales, starting one day post-viral challenge (Day 1) up to discharge from quarantine
• Peak daily symptom score: Individual maximum daily sum of Symptom score starting one day post-viral challenge (Day 1) up to the end of quarantine.
• Number (%) of participants with Grade 2 or higher symptoms

Objective: To assess the incidence of SARS-CoV-2 illness, in healthy volunteers, by inoculum dose
Endpoints: The incidence of:
• Upper Respiratory Tract illness (URT)
• Lower Respiratory Tract illness (LRT)
• Systemic illness (SI)
• Febrile illness (FI)
• Proportion of Subjects with Grade 3 symptoms on any occasion at any time from the last assessment on Day 0 to quarantine discharge
• Proportion of Subjects with Grade 2 or higher symptoms on any occasion at any time from the last assessment on Day 0 to quarantine discharge
• Proportion of Subjects with Grade 2 or higher Symptoms on two separate occasions at any time from the last assessment on Day 0 to quarantine discharge
• Proportion of Subjects with any symptom (grade >=1) on any occasion at any time from the last assessment on Day 0 to quarantine discharge
• Proportion of Subjects with any symptom (grade >=1) on two separate occasions at any time from the last assessment on Day 0 to quarantine discharge
